# Evaluation of efficacy of oxygen-enriched oil-based gel dressing in patients who underwent surgical repair of distal hypospadias: a prospective randomised clinical trial

**DOI:** 10.1007/s00345-020-03419-1

**Published:** 2020-08-27

**Authors:** Ciro Esposito, Fulvia Del Conte, Mariapina Cerulo, Vincenzo Coppola, Giovanni Esposito, Elisabetta Ricciardi, Felice Crocetto, Marco Castagnetti, Antonio Calignano, Maria Escolino

**Affiliations:** 1 Division of Pediatric Surgery, Federico II University of Naples, Via Pansini 5, 80131 Naples, Italy; 2 Department of Pharmacy, Federico II University of Naples, Naples, Italy; 3 Division of Pediatric Urology, Medical University of Padua, Padua, Italy

**Keywords:** Hypospadias, Dressing, Oxygen-enriched oil-based gel, Wound, Complications, Children

## Abstract

**Purpose:**

This study aimed to evaluate the efficacy of oxygen-enriched oil-based gel dressing on wound healing and postoperative outcome in children who underwent distal hypospadias repair.

**Methods:**

We included all patients with distal hypospadias, who underwent Snodgrass urethroplasty and preputioplasty over an 18-months period. The patients were randomized in two groups according to the type of medication: oxygen-enriched oil-based gel (G1) and hyaluronic acid cream (G2). After discharge, parents changed the dressing twice a day for 2–3 weeks postoperatively. The patients were evaluated at 7, 14, 21, 30, 60 and 180 postoperative days and thereafter annually.

**Results:**

One-hundred and fourteen patients (median age 18 months) were included in the study and randomized in two groups, each of 57 patients. The wound healing was significantly faster in G1 compared with G2 (*p* = 0.001). G1 reported significantly higher SWAS and modified HOPE scores compared with G2 (*p* = 0.001) at all steps of follow-up. No adverse skin reactions occurred. Foreskin dehiscence and re-operations rates were significantly lower in G1 compared with G2 (*p* = 0.001). Postoperative foreskin retractability was better in G1, with a significantly higher incidence of secondary phimosis in G2 (*p* = 0.001). The median treatment costs were significantly lower in G1 compared with G2 (*p* = 0.001).

**Conclusion:**

Postoperative dressing using oxygen-enriched oil-based gel was highly effective, promoting a faster wound healing in patients who underwent distal hypospadias repair. It reported a lower incidence of foreskin dehiscence and better foreskin retractability compared with the control group. It was cost-effective and clinically safe without allergy or intolerance to the product.

## Introduction

Hypospadias repair is one of the most common operations performed by pediatric urologists [1]. About 300 different surgical techniques have been previously described and used for hypospadias repair [2]. Probably, tubularized incised plate urethroplasty (TIPU), described by Snodgrass, is one of the most popular techniques adopted over the last 10–15 years [3, 4]. A recent meta-analysis about complication rates of the tubularized incised plate (TIP) repair reported fistula and re-operation rates of 5.7% and 4.5%, respectively, in primary distal cases whereas higher complication rates are seen with secondary and proximal repairs [5].

Analyzing the international literature, limited evidence is available regarding different aspects of surgical management of hypospadias, including details of operative technique, type of suture, indications for foreskin reconstruction, type and length of urinary diversion, and postoperative dressing [6].

Probably, one of the most controversial aspects of hypospadias surgery is the election of an appropriate wound dressing [1]. Multiple dressings have been previously described, using different types of materials such as silastic foam, elastic bands, glove finger, Tegaderm, Opsite, Cavicare and the more recent silicone-foam sheets (Mepilex and Allevyn) [7–14]. Nevertheless, there is no evidence in the current literature about the best method for postoperative dressing following hypospadias repair [1, 15]. An ideal hypospadias wound dressing should be cheap and non-allergenic. It should also be easy to apply and to remove, non-adherent to the incision; it should effectively absorb the leakages of the wound, produce an adequate compression of the penis, without damaging the blood circulation, thus preventing hematoma and edema formation and helping wound healing, protect against infections [11, 16, 17]. It also must keep its shape during the child’s movements, without limiting his normal activities [18].

Ozone (O_3_), in its topical form of ozonated oil, has been adopted in adults as wound-healing accelerator for different chronic wounds, such as trophic ulcer, ischemic ulcers and diabetic wounds, due to its bactericidal, antiviral and antifungal actions [19, 20]. Different experimental studies demonstrated that O_3_ may also act on acute wound healing directly or indirectly via collagen synthesis and fibroblast proliferation during granulation tissue formation and the early tissue remodeling phase of wound healing [21, 22].

Based on this experimental evidence, we hypothesized that the beneficial effects reported by ozone on the wound healing would also improve the outcome of hypospadias surgery. So, we decided to adopt this product for postoperative dressing in patients who underwent distal hypospadias repair and compare it with our standard dressing, represented by hyaluronic acid cream.

This study aimed to evaluate the efficacy of oxygen-enriched oil-based gel dressing on wound healing and postoperative outcome in children who underwent distal hypospadias repair.

## Materials and methods

We carried out a prospective single-blinded randomised clinical trial between March 2018 and September 2019. This study received the appropriate approval by the Institute Review Board (IRB) and the Ethics Committee. Written informed consent was obtained from all patients (or the legal guardian) to be recruited into the study.

### Study population

The study included all patients aged < 2 years with distal hypospadias, who received urethroplasty using Snodgrass technique and preputioplasty in our surgical unit. Patients with proximal hypospadias or patients with distal hypospadias aged > 2 years or toilet-trained at time of surgery were excluded from the study.

### Sample size and sampling method

The minimum sample size was calculated to allow to detect with a power of 80% and an alpha level of 0.05 an absolute difference in rates of at least 15%. This gave the minimum sample size to be 44 in each treatment arm, with a total number of 88. Adding an expected attrition rate of 30% (to account for eventual loss to follow‑up), the calculated sample size came to 114, randomised to 57 participants in each arm.

Randomization and patient allocation were performed using simple random sampling method, which entailed an equal number of ballot papers pre‑labelled with either oxygen-enriched oil-based gel (G1) or hyaluronic acid cream (G2), sealed in similarly opaque envelopes and picked before the surgical procedure.

### Operative technique

All the patients received urethroplasty according to Snodgrass technique and foreskin reconstruction. All the surgical procedures were performed by one experienced surgeon. Additionally, the same sutures and postoperative urinary diversion were adopted in all patients.

**Fig. 1:**
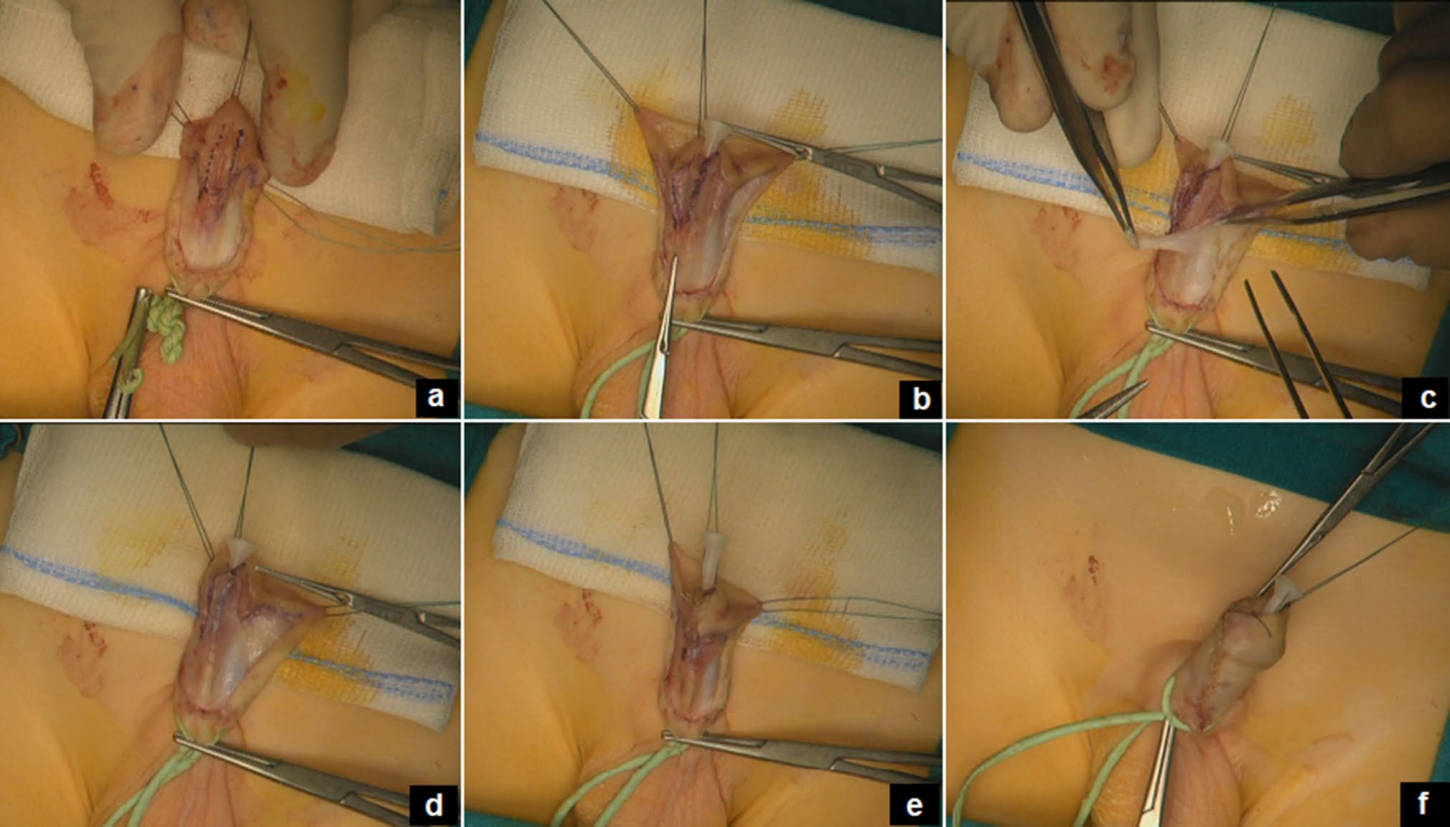
Steps of operative technique (TIPU): the urethral plate is incised (**a**) and tubularized over an 8 Fr stent (**b**); a ventral dartoic flap is isolated (**c**) and placed over the urethroplasty (**d**); the glanuloplasty is performed (**e**) and finally the foreskin is reconstructed (**f**)

**Fig. 2:**
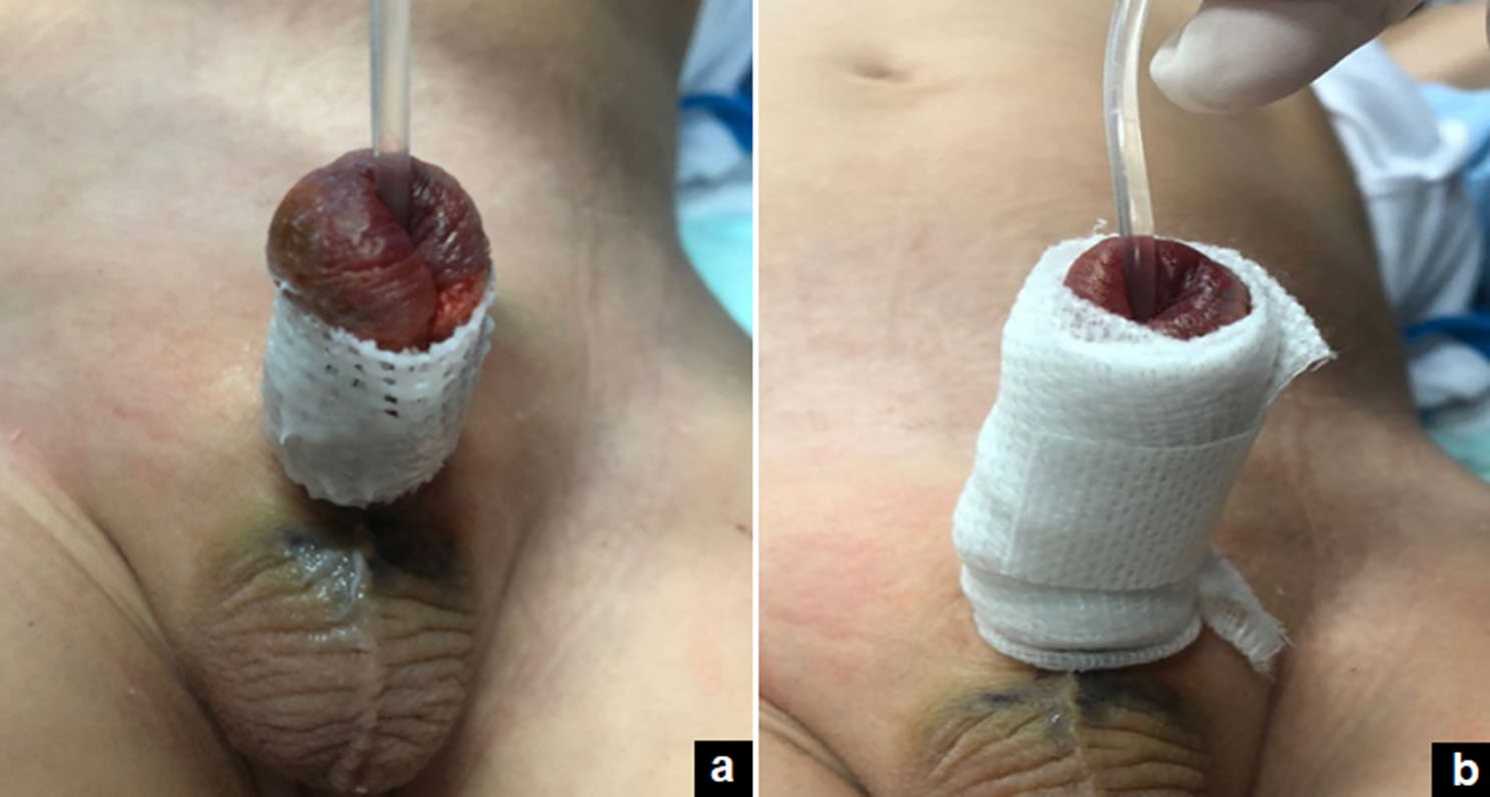
Postoperative dressing: a wet gauze, impregnated with oxygen-enriched oil-based gel or hyaluronic acid cream, is wrapped around the penis (**a**) and covered by an elastic net bandage (**b**)

After degloving the penis to remove chordees and correct the ventral curvature, the urethral plate was incised and tubularized over an 8 Fr stent. The urethroplasty was fashioned using a running suture of 6/0 monofilament polyglyconate suture followed by a second layer of interrupted stitches using the same suture. The new urethra was then covered with a large piece of ventral based vascularized subcutaneous dartos tissue, that was fixed to the new urethra using interrupted mattress stitches. Glanuloplasty was then performed using 5/0 polyglyconate interrupted sutures. Preputioplasty was finally performed and involved a three-layer closure consisting of the inner foreskin, middle dartos, and outer foreskin, that were approximated with 6/0 and 5/0 absorbable polyglyconate interrupted sutures (Fig. 1). All the patients had an 8 Fr Foley silicon catheter inside the urethra, and they were managed using a double diaper layer; the catheter was passed from a first to a second diaper through an opening, preventing the urine to be in touch with the dressing. After completing the surgical correction of the hypospadias, the area was washed with saline and dried with gauze. A layer of oxygen-enriched oil-based gel (G1) or hyaluronic acid cream (G2) was directly applied on a wet gauze composed by hyaluronic acid. This impregnated gauze was wrapped around the penis and subsequently covered by an elastic net bandage to obtain hemorrhage compressive effect (Fig. 2).

### Postoperative care

The first postoperative dressing was performed 48 h after surgery, the compressive effect was released and thereafter the dressing was repeated daily until the bladder catheter removal that occurred between the 5th and 7th postoperative day. During hospitalization, all the patients received antibiotic therapy (sulfamethoxazole/trimethoprim) at a dosage of 4 mg/kg (trimethoprim component) orally every 12 h and a single daily dose of probiotic [*L. rhamnosus* GG (ATCC 53103)] in the form of five drops containing 5 × 10^9^ colony-forming units (CFU), until the removal of the bladder catheter.

After hospital discharge, all parents were asked to apply topically the oxygen-enriched oil-based gel (G1) or the hyaluronic acid cream (G2) and a wet gauze wrapped around the penis without any bands during the diaper change twice a day for at least 2–3 weeks postoperatively. We provided the parents with the oxygen-enriched oil-based gel in a 5-ml sealed syringe or the hyaluronic acid cream tube without the external envelope indicating the name and composition of the product to ensure the blinding of the family to the dressing type. The parents were also encouraged to change the diaper frequently to avoid prolonged direct contact of the wound with the stools.

### Assessment of outcome parameters

The follow-up evaluations were performed by two independent pediatric surgeons not involved in the operation and blinded to the patient group. The follow-up schedule included a clinical control at 7, 14, 21, 30, 60, 180 days postoperatively and thereafter annually. At each control, the wound was observed and photographs of the penis were taken to document the evolution of the healing process and the aesthetic result. The functional and cosmetic results were objectively assessed using the modified Hypospadias Objective Penile Evaluation (HOPE) score (Table 1), that was previously described by our group [23]. The surgery was defined as successful in presence of a vertical slit-like appearance of the urethral meatus on the tip of the glans and a normal appearance of the reconstructed foreskin without scars, irregularity, or asymmetry and a straight penile axis without curvature or torsion. The wound healing was scored using the Southampton Wound Assessment Scale (SWAS) [24] and included normal healing (grade 0), normal healing with mild bruising or erythema (grade 1), presence of erythema plus other signs of inflammation (grade 2), clear or hemo-serous discharge (grade 3), or major complications such as pus (grade 4) and deep or severe wound infection with or without tissue breakdown or hematoma requiring aspiration (grade 5) (Table 2). The wound healing, used as endpoint in our study, was defined as a return to normal anatomic structure and appearance of the penis that included a fully differentiated and organized dermis and epidermis with an intact barrier function. The accurate observation of the wound allowed to distinguish between fibrinous exudate and pus (grade 4 SWAS) in the “yellow” lesion. In general, the “yellow” lesion is covered with tissue whose color can range from whitish to yellow or greenish. The different colors also correspond to different “contents” of bacterial load. A paler, lighter yellow color may indicate the presence of fibrin whereas a dark yellow/greenish color indicates the presence of infection and necrotic tissue. Furthermore, we carefully examined the peri-wound; if there was erythema, odor and any signs and symptoms, either local or systemic, of infection, we considered that we were most likely dealing with purulent drainage.

**Table 1:** Modified hypospadias objective penile evaluation (HOPE) score

Position meatus
Position 1—tip of glans (10 points)
Position 2—coronal (8 points)
Position 3—subcoronal (5 points)
Position 4—middle penis (3 points)
Position 5—tip of glans (1 point)
Shape meatus
Normal (10 points)
Slightly abnormal (7 points)
Moderately abnormal (4 points)
Severely abnormal (1 point)
Shape glans
Normal (10 points)
Slightly abnormal (7 points)
Moderately abnormal (4 points)
Severely abnormal (1 point)
Shape skin
Normal (10 points)
Slightly abnormal (7 points)
Moderately abnormal (4 points)
Severely abnormal (1 point)
Shape reconstructed prepuce
Normal (10 points)
Slightly irregular or asymmetric (7 points)
Moderately irregular or asymmetric (4 points)
Severely irregular or asymmetric (1 point)
Persistence of “dog-ears”
No (10 points)
Yes (3 points)
Retractability of reconstructed prepuce
Retractile foreskin (10 points)
Tight foreskin (3 points)
Torsion
0–30° (10 points)
30–50° (7 points)
50–70° (4 points)
> 70° (1 point)
Curvature in penile erection
0–30° (10 points)
30–50° (7 points)
50–70° (4 points)
> 70° (1 point)

**Table 2:** Southampton wound assessment scale (SWAS)

Grade	Appearance
0	Normal healing
1	Normal healing with
a	Some bruising
b	Considerable bruising
c	Mild erythema
2	Erythema plus other signs of inflammation:
a	At one point
b	Around sutures
c	Along wound
d	Around wound
3	Clear or hemoserous discharge:
a	At one point only (< 2 cm)
b	Along wound (> 2 cm)
c	Large volume
d	Prolonged (> 3 days)
*Major complication*	
4	Pus
a	At one point only (< 2 cm)
b	Along wound (> 2 cm)
5	Deep or severe wound infection with or without tissue breakdown; hematoma requiring aspiration

The evaluation of adverse reactions as skin irritability was categorized by the researcher observation as absent, limited to the foreskin, or extended to other areas. During the outpatient evaluation, the parents were also asked to describe the grade of the discomfort of their child with the use of dressing as “very troubled”, “troubled” or “not troubled”. As the assessment of “troubling” in a child is by its nature subjective, we tried to standardize this definition among the parents and we defined the child “troubled” in dealing with the dressing when he cried during the dressing change.

The grade of adhesiveness of the product was evaluated asking the parents if they found the product still adherent to the suture line at each diaper change. The retractability of the foreskin at > 30 days follow-up was also evaluated as an outcome parameter. The first retraction of the reconstructed foreskin was performed by the evaluating surgeon at mean 4 weeks postoperatively and if retractable parents were asked to continue foreskin retraction at home during daily hygienic care. The patients were also evaluated for the presence of postoperative complications including infections, foreskin dehiscence, meatal stenosis and urethrocutaneous fistula. Postoperative complications were graded according to Clavien-Dindo grading system [25]. We also analyzed and compared the costs of dressing between the two groups.

### Statistical analysis

Statistical analysis was carried out using the Statistical Package for Social Sciences (SPSS Inc., Chicago, Illinois, USA), version 13.0. Continuous variables were summarized and presented as median and interquartile range. The categorical variables were presented as absolute numbers and percentages. The demographic data were compared using the Student’s *t* test. The categorical variables were compared using *χ*² tests whereas the analysis of variance test was used to compare the subjective and objective assessment scores. Significance was defined as *p* < 0.05.

## Results

One-hundred and fourteen patients, with a median age of 18 months (interquartile range, IQR 12–30), were included in the study. All of them were not yet toilet-trained and wear a diaper at the time of surgery. The patients were randomized in two groups, each of 57 patients: the Treatment Group (G1) included patients treated using oxygen-enriched oil-based gel NOVOX ® (MOSS SpA, Lesa, Novara, Italy) and the Control Group (G2) included patients receiving the standard dressing using hyaluronic acid cream. The ozonized oil-based medication, adopted in G1, comes in an oily gel form in a 5-mL sealed syringe whereas the hyaluronic acid cream, adopted in G2, was aqueous based and not greasy.

There was no significant difference in the age distribution (*p* = 0.33), the proportion of hypospadias degree (*p* = 0.37), hospital stay (*p* = 0.55) and follow-up duration (*p* = 0.37) between the two groups. The patient’s baseline/demographics are summarized in Table 3.

**Table 3:** Patient’s baseline/demographics and outcome comparative analysis between G1 and G2

	G1 Oxygen-enriched oil-based gel *n = *57	G2 Hyaluronic acid cream *n = *57	*p* value
Median patients age, years (IQR)	17.5 (15–30)	18.5 (12–28)	0.33
Balanic hypospadias, *n* (%)	12/57 (21.1%)	9/57 (15.8%)	0.37
Coronal hypospadias, *n* (%)	39/57 (68.4%)	43/57 (75.4%)	0.37
Subcoronal hypospadias, *n* (%)	6/57 (10.5%)	5/57 (8.8%)	0.37
Median hospital stay, days (IQR)	7 (5–9)	7 (6–8)	0.55
Median follow-up, months (IQR)	14 (6–16)	16 (8–18)	0.37
Median indwelling bladder catheter, days (IQR)	5.8 (5–7)	6.1 (5–7)	0.33
Median wound healing time, days (IQR)	15.8 (11–18)	27.5 (17–35)	0.001
Adverse skin reaction to the product, *n* (%)	0	0	n/a
Patients’ discomfort with dressing No troubled, *n* (%) Troubled, *n* (%) Very troubled,* n* (%)	57/57 (100%) 0 0	24/57 (42.1%) 33/57 (57.9%) 0	0.001 0.001 n/a
Postoperative complications Urethrocutaneous fistula, *n* (%) Foreskin dehiscence, *n* (%) Meatal stenosis, *n* (%) Wound infection,* n* (%)	1/57 (1.7%) 0 0 0	3/57 (5.2%) 5/57 (8.7%) 0 0	0.57 0.001 n/a n/a
Total re–operations, *n* (%) Redo-urethroplasty (redo-TIPU), *n* (%) Urethrocutaneous fistula repair, *n* (%) Circumcision,* n* (%) Redo-preputioplasty,* n* (%)	1/57 (1.7%) 0 1/57 (1.7%) 0 0	8/57 (14.0%) 1/57 (1.7%) 2/57 (3.5%) 4/57 (7.1%) 1/57 (1.7%)	0.001 0.37 0.33 0.001 0.37
Foreskin retractability at >30 days follow-up Retractile, *n* (%) Phimosis,* n* (%): Preputial adhesions,* n* (%)	55/57 (96.5%) 0 2/57 (3.5%)	47/57 (82.4%) 4/57 (7.1%) 6/57 (10.5%)	0.001 0.001 0.001
Median cost 3-weeks treatment, eur (IQR)	46 (23–69)	54 (34–68)	0.001

*IQR* interquartile range, *n/a* not applicable

The analgesic therapy was administered in the first 24 h after surgery through an elastomeric infusion pump; thereafter, the analgesic drugs (paracetamol 15 mg/kg and tramadol 2 mg/kg every 8 h) were administered orally. The bladder catheter was removed between the 5th and 7th postoperative day in all patients, who were discharged the day following the catheter removal.

**Fig. 3:**
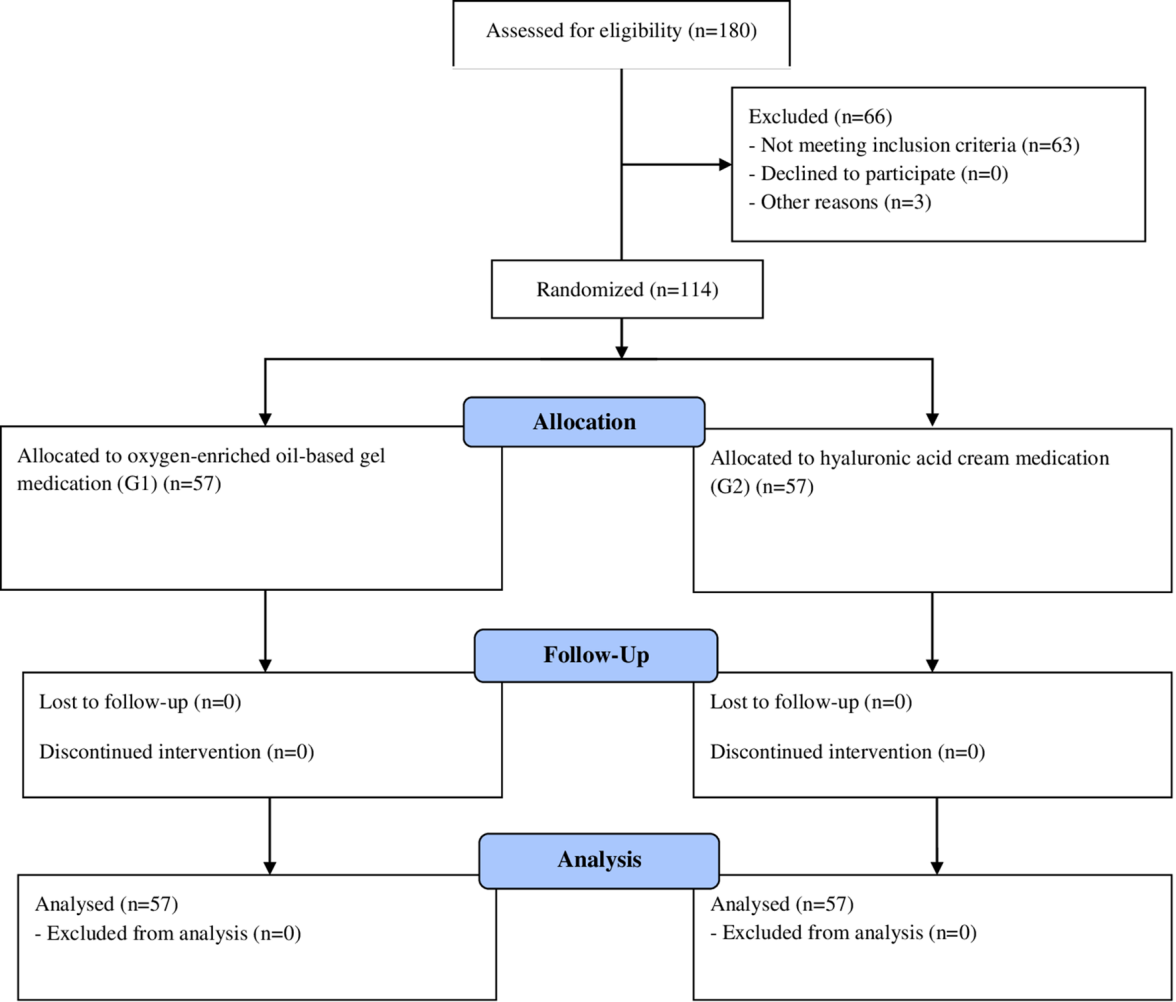
Patient allocation

**Fig. 4:**
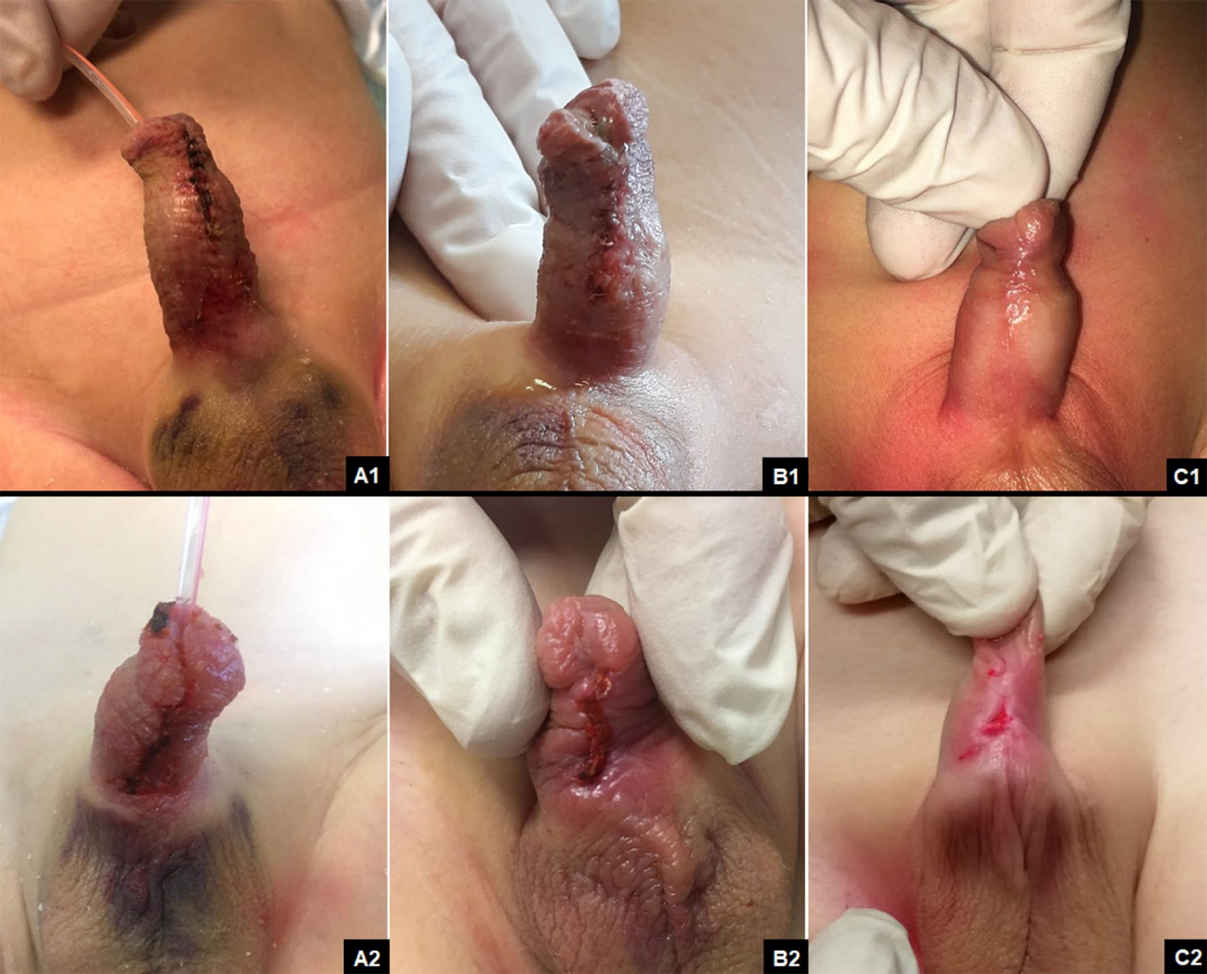
Wound healing comparison between oxygen-enriched oil-based gel at 7 days (**A1**), 14 days (**B1**) and 21 days (**C1**) follow-up and hyaluronic acid cream at 7 days (**A2**), 14 days (**B2**) and 21 days (**C2**) follow-up

No patients were lost to follow-up (Fig. 3). At the clinical evaluation, the wound healing time was significantly shorter in G1 compared with G2 [15.8 vs 27.5 days] (*p* = 0.001) (Fig. 4). Wound healing evaluation using SWAS reported a significantly higher rate of normal healing (≤ 1) in G1 compared with G2 at 7 days (78.9% vs 38.6%), 14 days (96.5% vs 57.9%), 21 days (100% vs 80.7%) and 30 days (100% vs 87.7%) follow-up (*p* = 0.001). No significant difference in SWAS scores was found between G1 and G2 (*p* = 0.33) at 60 days and 180 days follow-up. The functional and cosmetic outcome evaluation, using the modified HOPE score, showed significantly higher median scores in G1 compared with G2 at 7 days (82 vs 68), 14 days (83 vs 69), 21 days (87 vs 71), 30 days (90 vs 77), 60 days (90 vs 78) and 180 days (90 vs 81) follow-up (*p* = 0.001). Both SWAS and modified HOPE scores in each group are reported in Table 4.

**Table 4:** SWAS and modified HOPE scores in G1 and G2

	Southampton Wound Assessment Scale (SWAS)				*p* value
	G1 Oxygen-enriched oil-based gel *n* = 57		G2 Hyaluronic acid cream *n* = 57		
	≤ 1	> 1	≤ 1	> 1	
7 days follow-up	78.9% (45/57)	21.1% (12/57)	38.6% (22/57)	61.4% (35/57)	0.001
14 days follow-up	96.5% (55/57)	3.5% (2/57)	57.9% (33/57)	42.1% (24/57)	0.001
21 days follow-up	100% (57/57)	0	80.7% (46/57)	19.3% (11/57)	0.001
30 days follow-up	100% (57/57)	0	87.7% (50/57)	12.3% (7/57)	0.001
60 days follow-up	100% (57/57)	0	100% (57/57)	0	0.33
180 days follow-up	100% (57/57)	0	100% (57/57)	0	0.33

	Modified hypospadias objective penile evaluation (HOPE) score		
	Median score (IQR)	Median score (IQR)	*p* value
7 days follow-up	82 (70–83)	68 (58–70)	0.001
14 days follow-up	83 (70–87)	69 (62–76)	0.001
21 days follow-up	87 (70–90)	71 (69–87)	0.001
30 days follow-up	90 (80–90)	77 (68–87)	0.001
60 days follow-up	90 (80–90)	78 (69–83)	0.001
180 days follow-up	90 (83–90)	81 (71–90)	0.001

*IQR*  interquartile range

No adverse skin reactions to the product occurred in both groups. Finally, the grade of adhesiveness of the oxygen-enriched oil-based gel was higher compared with the hyaluronic acid cream; in fact, the parents of G1 patients stated that they always found the product still adherent to the suture line at each diaper change and it was extremely easy to remove the wet gauze that did not stick to any part of the penis whereas the parents of G2 patients reported that the hyaluronic acid cream was quickly absorbed by the skin and made more challenging the removal of the wet gauze, that remained more adherent to the penis. In fact, 33/57 (57.9%) G2 patients resulted significantly “troubled” with the use of the dressing whereas all G1 patients were “not troubled” and the wound was easily managed by parents at home.

The evaluation of foreskin retractability showed a significantly higher incidence of secondary phimosis and preputial adhesions in G2 (10/57, 17.6%) compared with G1 (2/57, 3.5%) (*p* = 0.001). All patients with postoperative phimosis or preputial adhesions were successfully treated by topical steroid application.

Regarding postoperative complications, no significant difference was found in regard to urethrocutaneous fistula rate between G1 [1/57 (1.8%)] and G2 [3/57 (5.2%)] (*p* = 0.57) (IIIb Clavien) whereas the incidence of foreskin dehiscence was significantly lower in G1 [0/57 (0%)] compared with G2 [5/57 (8.7%)] (*p* = 0.001) (IIIb Clavien). No wound infections were reported in both groups. Re-operations rate was also significantly lower in G1 [(1/57) 1.8%] compared with G2 [(8/57) 14.0%] (*p* = 0.001). Re-operations included urethrocutaneous fistula closure (*n* = 1) in G1 and redo-urethroplasty (redo-TIPU) (*n* = 1), urethrocutaneous fistula closure (*n* = 2), circumcision for preputial dehiscence (*n* = 4), and redo-preputioplasty for preputial skin fistula (*n* = 1) in G2.

Regarding the cost analysis, although the oxygen-enriched oil-based gel medication was more expensive compared with hyaluronic acid cream (23 vs 18 eur), this difference was overcome by the advantages related to the physical characteristics of the viscosity of the ozone oil allowing to adopt a very little amount of the product at each application, whereas the hyaluronic acid cream was aqueous based and not greasy and required a greater amount of the product at each use. We demonstrated the cost-effectiveness of ozone medication; in fact, to accomplish a 3-weeks postoperative treatment, G1 patients needed median two 5-mL syringes of the oxygen-enriched oil-based gel with a cost of 46 eur compared with G2 patients who needed median three 30-mL tubes of hyaluronic acid cream with a cost of 54 eur (*p* = 0.001).

The comparative outcome analysis between G1 and G2 is reported in Table 3.

## Discussion

Hypospadias surgery is one of the most common operations performed by pediatric urologists but several controversies still exist regarding the surgical techniques and also the postoperative management [1, 6]. Probably, the surgical dressing represents the greatest variable and source of the controversy of postoperative care [1]. The first debated point is the real need to use postoperative dressing in all hypospadias. Some authors reported that dressings were not necessary for all hypospadias surgeries and demonstrated that an absent hypospadias dressing did not compromise the outcome of reconstruction and did not increase postoperative complications or reoperations rate [26]. Hypospadias surgery is most often performed in children younger than 2 years of age, who are not toilet trained. In such patients, the presence of diaper is probably associated with an increased risk of wound contamination by stools and subsequent infection. We believe that a dressing should be always applied after hypospadias surgery, especially in children wearing the diaper, considering its protective function of mechanical barrier against tissues’ contamination and reduction of the edema caused by the surgical trauma. The second debated point is the type of dressing to be used following hypospadias surgery. An ideal dressing should present physical characteristics including elasticity, resistance and flexibility, and should provide an effective pressure on the wound [11]. The dressing should present minimal adverse reactions when in contact with tissues and it should be easy and painless to remove [16–18].

More recently, ozone (O_3_), in its topical form of ozonated oil, has been reported as an advanced clinical therapeutic agent for the treatment of both chronic and acute wounds in different surgical and medical specialties, with significant improvements in healing outcomes and healing time compared with the standard care [19–22]. We recently described the efficacy of the oxygen-enriched oil-based gel for wound healing in patients with hidradenitis suppurativa who underwent endoscopic treatment [27].

Wound healing is a multiphase process that consists of three overlapping but distinct stages: inflammation, new tissue formation and tissue remodeling. In the first 48 h after injury, different immune cells such as neutrophils, monocytes, and lymphocytes work together to prevent the bleeding and remove the dead tissues to balance the inflammation process and make the appropriate repair of the wound. In the next 2–10 days, new tissue formation is followed via cellular proliferation and migration of different cell types such as fibroblasts, keratinocytes, and endothelial cells. At this stage, fibroblasts play very important roles in the new tissue formation. The wound will increase the proliferation and migration of fibroblasts to promote the scar formation. In addition, fibroblasts can secrete many factors, such as matrix metallopeptidase-14 (MMP-14), basic fibroblast growth factor (bFGF), fibroblast growth factor-9 (FGF-9) to regulate the collagen homeostasis, angiogenesis, or other important functions to facilitate the wound healing. In addition, fibroblasts can differentiate into myofibroblasts, which produces an extracellular matrix and ultimately forms the mature scar. In 2–3 weeks after injury, the tissue remodeling process happens which may last for a year or more. At this stage, the entire processes activated by injury will wind down and cease while the activated cells will undergo apoptosis. Different cells (fibroblasts, macrophages, and endothelial cells) will secrete matrix metallopeptidase to remodel and strengthen the repaired tissues. Through these processes, the wound will be repaired. Experimental studies demonstrated that ozone can activate the fibroblasts and promote the migration and epithelial-mesenchymal transition (EMT) process in fibroblasts to facilitate the wound healing [28, 29]. The beneficial effects of ozone on the wound healing are due to the reduction of microbial infection, debridement effect, modulation of the inflammatory phase, stimulation to angiogenesis as well as biological and enzymatic reactions that favor the oxygen metabolism, improving the wound cicatrization. In surgical wounds, when the skin edges are approximated, the effect of ozone on the skin is due to its reaction with the polyunsaturated fatty acids and traces of water present in the upper layer of the dermis, generating reactive oxygen species (ROS) and lipo-oligopeptides, among which is H_2_O_2_ [19]. ROS are the most effective natural agents against antibiotic-resistant pathogens. Furthermore, ozone, by releasing oxygen (O_2_), activates the proliferation of fibroblasts and the synthesis of collagen fibers, hence the building of intercellular matrix with the consequent proliferation of keratinoblasts and acceleration of wound closure [19, 28].

Based upon this growing evidence, we decided to use the oxygen-enriched oil-based gel in patients who underwent distal hypospadias repair for at least 2–3 weeks postoperatively to include all the steps of the wound healing process. Long term topical application of the product, for a period ranging from 1 to 6 months, has also been recently described in different indications with very promising results [27, 30]. Our results showed a faster healing process and a significantly higher rate of normal healing (≤ 1), evaluated using SWAS, with no signs of inflammation such as bruising and erythema or edema, in the ozone group compared with the control group, at 7, 14, 21 and 30 days follow-up. Furthermore, a lower incidence of foreskin dehiscence was observed in the ozone group compared with the control group. This last finding may be due to the decreased postoperative inflammation and edema provided by oxygen-enriched oil-based gel, that reduced the tension in the sutured area and subsequent risk of foreskin dehiscence. Another beneficial effect was seen on the foreskin retractability; in fact, patients treated with ozone reported a significantly lower incidence of postoperative phimosis or preputial adhesions compared with the control patients. The better outcome of foreskin retractability may be probably due to the decreased edema and inflammation observed postoperatively. The decreased edema, that was observed after using the oxygen-enriched oil-based gel, may be explained by the experimental evidence that ozone can precociously suppress the inflammation of the injured tissues, thus reducing the edema, that is one of the earliest inflammatory signs [28]. Another key factor was to leave in place the compressive dressing, that was performed intra-operatively after hypospadias repair, for at least 48 h postoperatively to achieve an adequate contention of distension and limit the occurrence of postoperative edema. On the 3rd postoperative day, the compression was removed in all patients and the wound was treated daily with the oxygen-enriched oil-based gel.

Based upon our experience, we would outline some considerations and tips and tricks about the use of the oxygen-enriched oil-based gel for hypospadias dressing. The product has an unpleasant smell, that may be fastidious at beginning of treatment. It comes in an oily gel form in a 5-mL sealed syringe. At the first use, the gel is enough fluid and easy to apply; for the following uses, the product must be kept into the fridge, it becomes solid and it is more difficult to apply. So, after the first use, it should be put out of the fridge 5 min before each application to allow the product to become fluid at ambient temperature and easily applicable. The viscosity of the oily gel allowed the product to adhere to the wound without keeping it moist. These characteristics made the dressing painless and fast to perform at home with no troubles for the child and no excessive time losses for the parents. We also encouraged the parents to change the diaper frequently to avoid prolonged direct contact of the wound with the stools.

The treatment with ozone was also cost-effective; in fact, although the oxygen-enriched oil-based gel medication was more expensive compared with hyaluronic acid cream, this difference was overcome by the advantages related to the physical characteristics of the viscosity of the ozonized oily gel allowing to adopt a very little amount of the product at each application. We observed that the different density of the product was associated with different times of skin absorption. In fact, the higher viscosity of the oxygen-enriched oil-based gel was associated with longer skin absorption time and consequently lower consumption of the product and lower costs. Furthermore, the viscosity of the oily gel made the dressing change more comfortable for the child. Based upon our experience, we believe that the substrate was crucial to boost up the efficacy of the active ingredient.

The study is not without limitations. First, our study was limited to distal hypospadias repair to eliminate the possible confounding factors related to the differences regarding the type of surgical technique, anatomic characteristics, postoperative management and postoperative complications rate associated with proximal hypospadias repair. However, this characteristic of our study limits the generalizability of the results. Another limitation of our study was the limited follow-up period. A longer follow‑up and a larger series including also patients with proximal hypospadias are required to validate these preliminary results.

In conclusion, based upon these preliminary results, postoperative dressing using oxygen-enriched oil-based gel was highly effective, promoting a faster wound healing in patients who underwent distal hypospadias repair. Furthermore, the wound treated with the oxygen-enriched oil-based gel was associated with better postoperative outcome including a lower incidence of foreskin dehiscence and better foreskin retractability compared with the control group. It was also cost-effective and clinically safe since no patients in our series suffered from allergy or intolerance to the product.
